# Recent Advances in the Application Peptide and Peptoid in Diagnosis Biomarkers of Alzheimer’s Disease in Blood

**DOI:** 10.3389/fnmol.2021.778955

**Published:** 2021-12-23

**Authors:** Yuxin Guo, Zhiyuan Hu, Zihua Wang

**Affiliations:** ^1^CAS Key Laboratory of Standardization and Measurement for Nanotechnology, CAS Key Laboratory for Biomedical Effects of Nanomaterials and Nanosafety, CAS Center for Excellence in Nanoscience, National Center for Nanoscience and Technology, Beijing, China; ^2^University of Chinese Academy of Sciences, Beijing, China; ^3^Fujian Provincial Key Laboratory of Brain Aging and Neurodegenerative Diseases, School of Basic Medical Sciences, Fujian Medical University, Fuzhou, China; ^4^School of Nanoscience and Technology, Sino-Danish College, University of Chinese Academy of Sciences, Beijing, China; ^5^School of Chemical Engineering and Pharmacy, Wuhan Institute of Technology, Wuhan, China

**Keywords:** Alzheimer’s disease, blood biomarkers, diagnosis, peptide, peptoid

## Abstract

Alzheimer’s disease (AD) is one of the most common neurodegenerative diseases with irreversible damage of the brain and a continuous pathophysiological process. Early detection and accurate diagnosis are essential for the early intervention of AD. Precise detection of blood biomarkers related to AD could provide a shortcut to identifying early-stage patients before symptoms. In recent years, targeting peptides or peptoids have been chosen as recognition elements in nano-sensors or fluorescence detection to increase the targeting specificity, while peptide-based probes were also developed considering their specific advantages. Peptide-based sensors and probes have been developed according to different strategies, such as natural receptors, high-throughput screening, or artificial design for AD detection. This review will briefly summarize the recent developments and trends of AD diagnosis platforms based on peptide and peptoid as recognition elements and provide insights into the application of peptide and peptoid with different sources and characteristics in the diagnosis of AD biomarkers.

## Introduction

Alzheimer’s disease (AD) is one of the most common neurodegenerative diseases, leading to a rapid decline in cognitive impairment. As a result of the aging population and the improvement of life quality, the number of people affected by AD will increase several times in the next few decades, from about 40 million at present to more than 100 million in 2050 (Scheltens et al., 2016; Hajipour et al., 2017; Hampel et al., 2018; Jia et al., 2018). AD is a chronic disease that gradually progresses over time. Before clinical symptoms appear, the patients have already had physiological changes in their brains and changes in the cerebrospinal fluid (CSF) concentration of related proteins (Blennow and Zetterberg, 2018). Diagnosis in the early stages means less disease progresses, conducive to disease intervention (Nordberg, 2015). Therefore, there is an urgent need for ideal AD diagnostic technology, which is non-invasive or minimally invasive, with high accuracy and facilitates large-scale screening to improve early diagnosis, primary care, and precision medicine (Hampel et al., 2018).

Currently, nano-biosensors and molecular probes have been developed in the field of biomarkers detection and molecular imaging with the aim of AD diagnosis (Kaushik et al., 2016; Jun et al., 2019; Yang et al., 2019; Bilal et al., 2020; Hanif et al., 2021). Due to the inherent characteristics of a high surface-area-to-volume ratio, targeting ligand surface modification and higher blood-brain barrier (BBB) permeability, nanomaterials have great potential in fast, selective and sensitive detection of biomarker (Gopalan et al., 2020). At the present time, the most widely used methods for AD fluid biomarkers detection by enzyme-linked immunosorbent assay (ELISA) based on commercial monoclonal antibodies (mAbs). However, due to the complicated production and high cost, but there are some limitations using traditional antibodies as detection receptors. The new emerging synthetic and bio-mimetic receptors such as peptide and small molecules have alternative bio-analytical approaches for AD biomarkers detection (Scarano et al., 2016).

In particular, peptide-based materials have recently been widely used in imaging, disease detection and drug delivery systems (Tweedle, 2009; Wang and Hu, 2019). Peptide and peptoid probes have also been proven to have great potential in detecting and diagnosing AD, which benefits from their small molecular weight, strong affinity and specificity, low immunogenicity, easy synthesis and modification (Baig et al., 2018b). On the one hand, as a recognition element, it binds explicitly to disease-related proteins to achieve high sensitivity and specificity, rapid, low-cost, and reliable biomarker detection (Baig et al., 2018a; Zafar et al., 2021). Due to their large number and diverse structures, it is relatively easy to find peptide ligands specifically binding to Aβ42 biomarkers compare with monoclonal antibody. On the other hand, peptide-based materials are promising to be used as Magnetic Resonance Imaging (MRI) contrast agents and imaging probes to visualize Aβ biomarkers to diagnose AD effectively (Gopalan et al., 2020). In addition, a peptide can self-assemble through intramolecular or intermolecular non-covalent bonds (such as electrostatic interaction, hydrogen bonding, van der Waals force, and π-π stacking, etc.) to form different nanostructures such as nanobelts, nanofibers, and so on (Wei et al., 2017). Peptide-based nanomaterials can provide enhanced surface area and binding sites to increase the accumulation of signal probes and bind particular peptide domains to display specific functions (Wei et al., 2017; Qi et al., 2018). As a result, self-assembled peptide nanomaterials have become one of the signal amplification strategies of biosensors in AD detection and imaging.

In this review, examples of the application of peptide-based materials to AD detection will be reported according to different sources and functions, and the method adopted for their selection and synthesis as well:

• Peptides from natural receptors or pronucleion peptides: stable artificial tiny receptors can be synthesized from known innate receptor sequences. For AD biomarkers, the most typical are cellular prion protein (PrPC), a natural Aβ oligomer receptor, and KLVFF (Aβ amino acid residues 16–20), the core area responsible for Aβ self-association and aggregation.
• Peptides from combinatorial peptide libraries: a variety of combinatorial libraries have been used for affinity peptide screening targeting AD biomarkers. Peptide library screening methods usually require little or no prior knowledge of the required sequence or structural features.
• Peptoid: peptoid is a peptide mimic with better protease resistance, stability, and enhanced pharmacological properties. Besides, peptoids have been designed as nanosheets with cyclic structures on the surface, mimicking the structure of antibodies.
• Enzyme-responsive peptides: as the Beta-secretase 1 (BACE1) enzyme also plays a vital role in AD pathology, peptides have been designed and synthesized as enzyme-responsive substrates to detect enzyme activity.

## Diagnosis of Alzheimer’s Disease

At present, AD is considered to be a continuous process of neurological decline, which can be identified and staged by a combination of neuropathological findings and biomarkers in the body (Khoury and Ghossoub, 2019). Traditional AD biomarkers mainly include Aβ42, Aβ42/Aβ40, p-tau, and t-tau in CSF and blood. The accumulation and deposition of Aβ in the brain is a critical factor in the pathogenesis of AD and is used as a biomarker of AD (Selkoe et al., 1994; Nakamura et al., 2018). However, the absolute Aβ42 level is affected by many factors, and individual differences are apparent (Milà-Alomà et al., 2019). Since the concentration of Aβ40 in CSF is ten times higher than that of Aβ42 and its level usually does not change in AD (Lewczuk et al., 2017), the use of Aβ42/Aβ40 as an AD biomarker may be more reliable than the absolute value of Aβ42, that the AUC in the blood can reach 0.89 (Ovod et al., 2017). Hyperphosphorylation of tau protein leading to neurofibrillary tangles is another significant pathological sign of AD (Goedert, 1993; Alonso et al., 2001). In the brain of AD patients, the phosphorylation level is 3–4 times that of normal tau protein (Schöll et al., 2019). Among Tau-related biomarkers, t-tau has also been found to be elevated in a variety of neurological diseases, so it is considered to be a related biomarker of neuronal damage and has low specificity for AD (Schöll et al., 2019). As p-tau (especially p-tau181) is present at normal levels in most other neurodegenerative diseases, p-tau has higher sensitivity and specificity for differential diagnosis of AD and other diseases (Janelidze et al., 2020; Thijssen et al., 2020; Brickman et al., 2021). The high levels of p-tau181 in plasma and CSF may reflect early Tau abnormalities that predate tau-PET abnormalities. In addition, some new potential AD biomarkers are being studied. BACE1 is a key enzyme that initiates the formation of Aβ peptides, and studies have shown that the concentration and activity of BACE1 increase in patients with AD and Mild Cognitive Impairment (MCI) (Jankowsky et al., 2004; Zetterberg et al., 2008). In addition to senile plaques and neurofibrillary tangles (NFTs), neurodegeneration and synapse loss are also inevitable in AD and increase with the progress of AD, which is manifested by the existence of a large number of dystrophic axons and dendrites surrounded by activated glial cells in the AD brain (Serrano-Pozo et al., 2011; Park et al., 2020). Neurofilament light chain (NfL) has been shown to be a biomarker of axon damage (Brureau et al., 2017). The destruction of the axon membrane releases NfL into the interstitial fluid, and finally into CSF and blood (Petzold, 2005). Many studies have shown that CSF and blood NfL levels increase with the progression of AD disease, and serum/plasma and cerebrospinal fluid NfL concentrations are highly correlated (Mattsson et al., 2017; Khalil et al., 2018; Preische et al., 2019). NfL has poor specificity for AD, but if combined with other indicators, it still has a high differential diagnosis value (Ashton et al., 2021; Cullen et al., 2021). Plasma NfL concentrations are increased in 13 neurodegenerative disorders and is not specific to AD.

Currently, AD diagnostic biomarkers include imaging, cerebrospinal fluid (CSF) detection and blood biomarkers. CSF biomarkers have high sensitivity and specificity and have been included in the diagnostic criteria for AD and used as biomarkers for the differential diagnosis of other types of dementia (Bjerke and Engelborghs, 2018). CSF is the most reliable and accurate biological fluid used for AD biomarker evaluation since CSF directly interacts with the extracellular space in the brain to reflect related biochemical/pathological changes (Blennow et al., 2015). However, the CSF extraction process is complex and invasive (CSF extraction requires professional equipment and lumbar puncture, which is not easy to be repeated many times), limiting its application in AD and MCI progression monitoring. Compared with CSF, the blood-based biomarkers of AD provide a cost- and time-effective way to enhance the utility of CSF (Henriksen et al., 2014; Dubois et al., 2016; Patricia et al., 2020). Because blood testing is clinical routines globally, allowing the use of existing systems to collect and process samples, blood-based AD biomarkers can meet the requirements of primary care settings to widely screen a large number of people (Hampel et al., 2018; Zou et al., 2020; Hansson, 2021). At the same time, blood biomarkers are conducive to multiple sampling and longitudinal studies. Blood-based testing would be widely available, easy and rapid to perform, and economical (Schneider et al., 2009; Crunkhorn, 2018; Hampel et al., 2018). Research on changes in biomarkers over time may show better results in AD screening and diagnosis (Weston et al., 2019; Moscoso et al., 2021). However, blood-based biomarkers have ultra-low concentrations (usually only a few to tens of pg/mL) and require ultra-sensitive sensors and analytical techniques to detect them (Andreasson et al., 2016). A recent study used immunoprecipitation-mass spectrometry (IP-MS) technology to score the ratio of APP669–711 to Aβ42 comprehensively and the ratio of Aβ40/Aβ42 to define whether the patient was AD positive or negative with 90% accuracy, further verifying the feasibility of using blood biomarkers to detect AD (Nakamura et al., 2018). AD-related imaging biomarkers mainly include MRI and Positron Emission Tomography (PET) diagnosis, which respectively reveal brain atrophy and the accumulation of amyloid, tau and other proteins in the brain (van Oostveen and de Lange, 2021). In addition to some tools and technologies currently in clinical trials, with the rapid development of optical probes, optical imaging not only has been used for fluorescence detection and imaging *in vitro* but also has become a reliable *in vivo* imaging tool (Arora et al., 2020).

## Peptide and Peptoid in Alzheimer’s Disease Diagnosis

### Peptides From Natural Receptors or Pronucleon Peptides

Synthesizing stable artificial small receptors from known natural receptor sequences is one of the common methods to obtain targeted peptides. For AD biomarkers, cellular prion protein (PrP*^C^*) is a high-affinity receptor specifically binding to Aβ oligomer (AβO), while not significantly binding to Aβ monomers or fibrils (Lauren et al., 2009; Chen et al., 2010). The core region of PrP*^C^* interacting with AβOs is PrP_95–110_, and the amino acid sequence is THSQWNKPSKPKTNMK, which is located in the unstructured N-terminal region of PrP*^C^*. The peptide has been used as a recognition element in a variety of electrochemical sensing technologies such as square wave voltammetry (SWV), electrochemical impedance spectroscopy (EIS), linear sweep voltammetry (LSV) and differential pulse voltammetry (DPV), to achieve high-sensitivity detection of AD biomarkers and signal amplification, with the potential for CSF and blood detection (Table 1).

**TABLE 1:** Electrochemical biosensor based on Aβ peptides.

Method	Electrode	Identify element	Detection range	LOD	References
EIS	Screen printing gold electrode	PrP_95–110_	1 pM–1 μM	0.5 pM	Rushworth et al., 2014
EIS	Gold electrode	PrP*^C^*-AuNPs	0.1 nM–0.2 μM	45 pM	Xia et al., 2017
LSV	Gold electrode	Ad-PrP_95–110_-AgNPs	0.01–200 nM	6 pM	Xing et al., 2017
DPV	Gold electrode	PrP_95–110_	3–7000 pg/mL	0.2 pg/mL	Negahdary and Heli, 2019
SWV	Gold electrode	CP4-PrP_95–110_, C16-GGG-PrP_95–110_-Fc	0.005–5 μM	0.6 nM	Huang et al., 2021
SWV	Gold electrode	KLVFFEEEEEE	3.3–3300 pg/mL	1 pg/mL	Zhang et al., 2019

Rushworth et al. (2014) have conjugated the biotinylated PrP95–110 to a polymer-functionalized screen printing gold electrode through a biotin/Avidin bridge to construct a label-free electrical impedimetric biosensor. As the combination of AβO and various metal cations increases the surface current density of the sensor, the impedance decreases with the increase of AβO concentration with a linear range of 1 pM–1 μM, and a LOD of 0.5 pM. Xia et al. (2017) used AβO and AuNPs competitively combined with PrP95–110 fixed on the surface of the gold electrode to prevent AuNPs from accumulating on the surface of the electrode, thereby increasing the impedance. The charge transfer resistance is proportional to the Aβ concentration in the range of 0.1 nM–0.2 μM, and the detection limit is determined to be 45 pM (Xia et al., 2017). According to a similar principle, Xing et al. (2017) constructed an electrochemical sensor based on peptide-induced AgNPs aggregation with a detection range of 0.01–200 nM and a LOD of 6 pM. The method has been proved can be applied to the analysis of AβO in serum and artificial cerebrospinal fluid (aCSF) (Xing et al., 2017). Negahdary and Heli (2019) electrodeposited microporous gold nanostructures with high surface area to immobilize PrP95–110 with high surface concentration to amplify the electrical signal. Using ferrous/ferricyanide as the redox probe, the peak current of DPV obtained is linear with the concentration of Aβ in the range of 3–7000 pg/mL, and the detection limit is 0.2 pg/mL (Negahdary and Heli, 2019). Huang et al. (2021) proposed a new signal amplification strategy based on in-situ peptide self-assembly. They designed a cysteine-containing peptide CP4-PrP (95–110) immobilized on the surface of a gold electrode to capture AβO, and another ferrocene-coupled peptide C16-GGG-PrP (95–110)-Fc to identify the captured AβO. The C16 hydrophobic domain initiates the peptide self-assembly *in situ*, thereby generating the accumulation of ferrocene as an electroactive reporter and obtaining an amplified electrochemical signal. The SWV peak intensity of the sandwich electrochemical sensor thus constructed increases linearly with the concentration of AβO, ranging from 0.005 to 5 μM. The LOD is 0.6 nM, and the signal-to-noise ratio is 3. Zhang et al. (2019) developed an electrochemical sensor to evaluate the neurodegenerative ability of Aβ secreted by platelets in forming and catalyzing oxidative cross-linking. The detection range is 3.3–3300 pg/mL, LOD < 1 pg/mL. Li et al. (2012) constructed a peptide-based electrochemical biosensor for Aβ 42 soluble oligomer assay, and the concentration range from 480 pM to 12 nM. Matharu et al. (2015) was designed and synthesized an MRI probe R2 [Gd-(DOTA)Grffvlkrrrrrr-NH2] combining Aβ42 fragment with cell-penetrating peptide to obtain high resolution images of amyloid plaques in AD model mice. This potential peptide MRI contrast agents may give rise to lower background staining as they are hydrophilic in character, containing more clustered positively charged side chains, and chelated gadolinium ion (Matharu et al., 2015).

**FIGURE 1:**
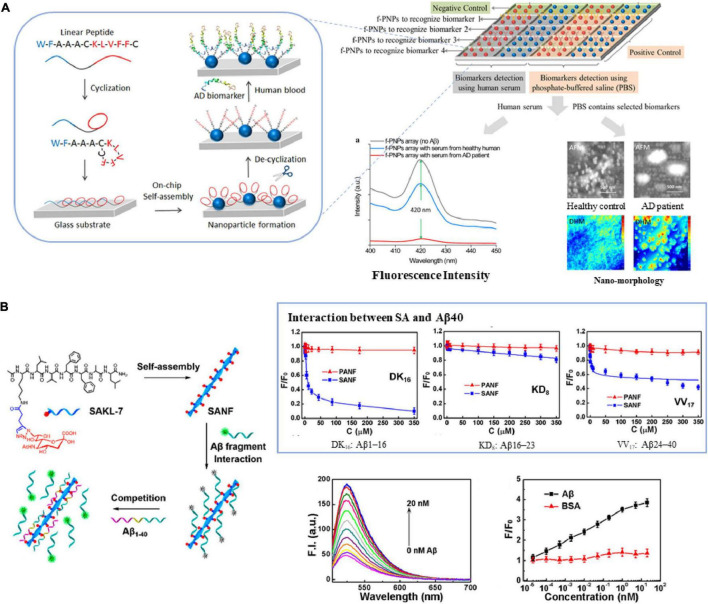
The core region KLVFF of Aβ is used as **(A)** recognition element of biosensors (Sun et al., 2018), **(B)** carrier for signal amplification (Lei et al., 2019). Reproduced with permission.

Studies have shown that KLVFF (amino acid residues 16–20), the central region of Aβ, is responsible for its polymerization and aggregation (Tjernberg et al., 1996). The sequence and its derived peptides are often used to selectively bind amyloid, thus developing various biosensing technologies, such as fluorescence assays, electrochemical biosensors, and immunoassays. Pradhan et al. (2015) used the aggregation-induced emission (AIE) molecule and peptide RGKLVFFGR, composed of the core region KLVFF and two terminal solubilizing components (RG–/–GR), to construct a fluorescent probe specifically binding to Aβ fibrils, which has a high signal-to-noise ratio and is not affected by traditional fluorescence quenchers. Sun et al. (2018) created a personalized array of lab-on-chip fluorescent peptide nanoparticles (f-PNPs) to detect multiple biomarkers in human blood samples. Taking Aβ as an example, the sequence of f-PNPs is WFAAACKLVFFC: KLVFF is used to bind to Aβ polypeptides, WF self-assembles into nanostructures, and cysteine cyclizes to protect the recognition sequence during self-assembly (Figure 1A). The change of fluorescence intensity and the morphological change of nanoparticles can significantly distinguish AD patients from healthy controls. Similarly, Liu et al. (2021) used KLVFF and Fmoc-KLVFF to self-assemble into f-PNPs through zinc coordination and π-π stacking, which can specifically detect Aβ aggregates (oligomers and fibrils) in the range of 10^–4^-10^–10^ mg/mL, and have the potential to be used in blood sample detection of Aβ. Matveeva et al. (2017) proposed a new non-antibody detection method based on pronucleon peptides to capture Aβ oligomer called PLISA, with a similar principle to ELISA. The detection limit of PLISA is 0.35–1.5 pM, and its monomer cross-reactivity (monomer/oligomer percentage) is much lower than ELISA.

Due to its self-assembly capability, structural controllability and good biocompatibility, the sequence KLVFF is also used as a carrier for molecular probes or recognition elements to achieve the purpose of signal amplification. Lei et al. (2019) coupled sialic acid (SA) to the KLVFFAL (KL-7) sequence, and it self-assembled to form nanofibers to obtain high-density SA on the surface. They used the probe to study the interaction between SA and Aβ, as well as Aβ aggregation. The linear range is 2 × 10^–5^–2 × 10^1^ nM, and the detection limit is 3.8 × 10^–4^ nM (Figure 1B). Subsequently, they used KL-7 loaded with Zn^2+^ specific dye AQZ and selective Cu^2+^-responsive near-infrared quantum dots (NIR QD) to achieve multicolor imaging of the ratio of Zn^2+^ to Cu^2+^ in live cells and zebrafish (Lei et al., 2020).

**FIGURE 2:**
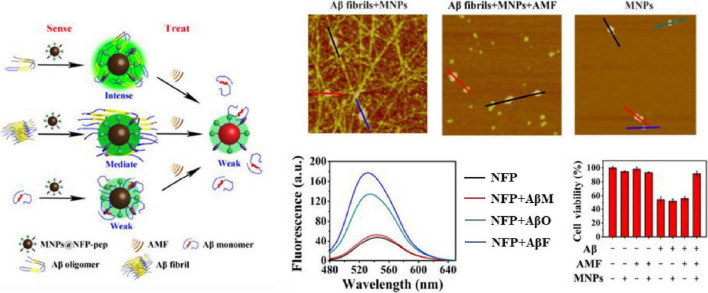
The core region KLVFF of Aβ is used as a binding element for Aβ targeting inhibitors (Du et al., 2018). Reproduced with permission.

In addition, this short peptide is also used as a “binding element” for the design of Aβ targeting inhibitors. Li et al. (2013) synthesized polyoxometalate (POM)-peptide mixed particles as a bifunctional Aβ inhibitor, which has an enhanced inhibitory effect on amyloid aggregation in the cerebrospinal fluid of mice. Du et al. (2018) synthesized a kind of magnetic nanoparticles (MNPs) modified with naphthimide-based fluorescent probes (NFP) and KLVFF peptides to improve the ability to recognize Aβ oligomers and penetrate BBB. While MNPs are exposed to an alternating magnetic field (AMF), Aβ aggregates decompose, the fluorescence of NFP is weakened and the morphological changes of Aβ can be monitored in real-time to realize the integration of diagnosis and treatment (Figure 2).

### Peptides From Combinatorial Peptide Libraries

Combinatorial peptide libraries are often used for screening to identify valuable components, such as ligands and drug candidates (Gray and Brown, 2014). The main advantage of combinatorial peptide library screening is to generate many binding peptides from an utterly random library, which neither contains any designed peptide structure nor the elements of the planned construction. Unbiased screening can generally identify groups of peptides with different binding motifs. Therefore, peptide libraries provide an opportunity to discover previously unknown binding epitopes and motifs.

Phage display is one of the most commonly used methods to identify specific peptide ligands and is widely used to enhance the active targeting of nano-drug delivery vehicles, imaging probes, or as a recognition element for detection technologies (Wu et al., 2016). Phage display is to insert the DNA sequence encoding the random peptide library into the DNA of the phage coat protein. When the phage is assembled, the protein-peptide fusion is expressed and integrated on the surface of the phage so as to screen the peptide sequence that binds to the target of interest (Pande et al., 2010). Phage display libraries generally can contain 10^8^ to 10^9^ different peptide sequences, including cyclic peptides. Many studies have been using phage display for peptide selection *in vitro* or *in vivo* for AD detection, imaging, or drug delivery (Table 2).

**TABLE 2:** Aβ-targeting peptides identified from phage-display peptide libraries.

Biopanning	Sequence	Binding affinity	Application	References
Aβ42	CIPLPFYNC(PHO) CFRHMTEQC(PHI)	2.2 × 10^–10^ M 5.45 × 10^–10^ M	Detection of Aβ42 (Lee et al., 2019)	Larbanoix et al., 2010
Aβ42	LIAIMA(Pep1) IFALMG (Pep2)	2.63 × 10^–6^ M 1.11 × 10^–6^ M	SPECT imaging agent (Jokar et al., 2020)	Larbanoix et al., 2011
Recombinant Aβ42	SGVYKVAYDWQH SPHLHTSSPWER	10.09 ± 1.67 μM 1.93 ± 0.11 μM	–	Kim S.-H. et al., 2020
Plasmas	HMRQGMA (AD#1) DGARHGA (Con#1)	–	Early diagnosis of AD	Chen et al., 2019
*In vivo*	TPSYDTYAELR PMKSHTN TGNYKALHPHNG	–	*In vivo* optical imaging	Li et al., 2011, 2015; Yang et al., 2021
soluble Aβ42	RFRK	4.5 ± 0.5 × 10^–5^ M	Inhibit the formation of soluble Aβ42 oligomer	Kawasaki et al., 2011
D-Aβ(1–42)	QSHYRHISPAQV	0.4 μM	Probes for *in vivo* detection of Aβ	Wiesehan et al., 2003
D-enantiomeric PHF6 Fibril	D-TTSLQMRLYYPP D-APTLLRLHSLGA	–	Tau aggregation inhibitor	Dammers et al., 2016
D-enantiomeric PHF6 Fibril	NITMNSRRRRNH	–	Prohibit the formation of PHF6 fibrils	Zhang et al., 2020

**FIGURE 3:**
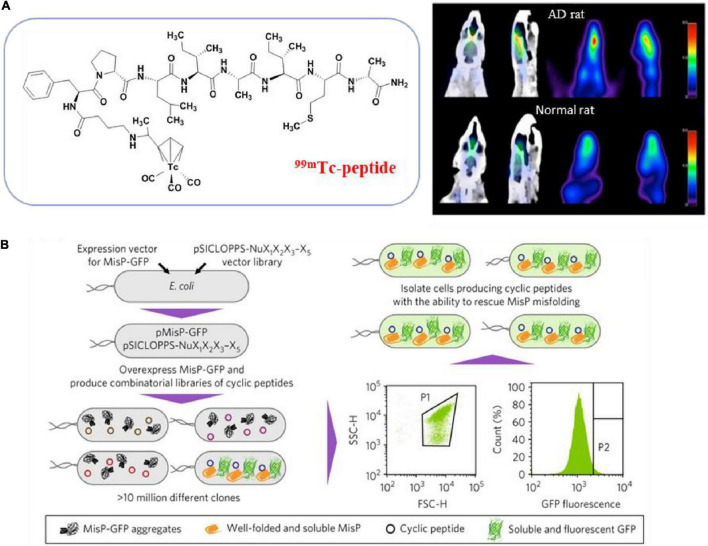
Peptides from phage display library screening methods. **(A)** Transform the peptide obtained from phage display screening to get SPECT imaging agent (Jokar et al., 2020); **(B)** schematic overview of the strategy for bacterial library screening used FACS (Matis et al., 2017). Reproduced with permission. Copyright 2017, Springer Nature.

Larbanoix et al. (2010, 2011) used phage libraries to screen Aβ-specific binding short peptides *in vitro*, and sequenced them to obtain four peptide sequences PHO, PHI, Pep1 and Pep2 with picomolar affinity. Some peptide-modified magnetic resonance imaging contrast agents can cross the blood-brain barrier and proved to be good candidates for Aβ42 imaging *in vivo*. Lee et al. (2019) used PHO and PHI peptides to design a polyvalent-directed peptide polymer (PDPP), aiming to enhance binding sensitivity and specificity by synergistically binding multiple target sites. And then, they modified it on the nanoporous zinc oxide with a high surface area to further improve the binding sensitivity and realize the detection of Aβ42 in CSF; the LOD can reach 12 ag/mL. Jokar et al. (2020) replaced all L-amino acids with D-type amino acids on the basis of Pep1, capped the C-terminus with an amide bond, and added D-proline and D-phenylalanine to the N-terminus, thus reducing the sensitivity to proteases, increasing brain uptake and inhibiting the formation of Aβ aggregates (Figure 3A; Jokar et al., 2020). They successfully synthesized ^99m^Tc-Cp-GABA-D-(FPLIAIMA)-NH2 as a potential SPECT imaging agent for early detection of Aβ plaques in the brains of AD patients. In addition to the use of purified receptors for biopanning, Chen et al. (2019) chose to use the plasma of AD patients and healthy controls to obtain AD-specific peptide AD#1 and control-specific peptide Con#1. Among them, AD#1 is combined with recombinant human YKL-40 protein in experiments *in vitro* (Chen et al., 2019). This screening method may be beneficial to obtain new valuable potential markers.

In recent years, phage display *in vivo* has received more and more attention because it completely replicates the natural physiological environment in the body and has the ability to screen peptides that target specific tissues (Andrieu et al., 2019). In this technique, the phage library is injected intravenously into live mice, rats and even humans, and then the phage is recovered from the tissues of interest. Due to the tight connection of the BBB and the efflux transport system, the delivery of drugs to the brain is severely restricted. *In vivo* phage technology facilitates screening peptides that can cross the BBB and blood-cerebrospinal fluid barrier (BCSFB). Li et al. (2011, 2015) successively used this technology to recover phage from rat cerebrospinal fluid to obtain several peptide sequences and verified their ability to pass through BBB or BCBFB by *in vivo* imaging analysis (Yang et al., 2021). They have the potential to construct drug delivery systems or imaging probes targeting the brain.

Because *E. coli* is easy to handle and multiplies, bacteria are also often used to display peptide libraries. In the *E. coli* library, the peptide gene is integrated into the membrane flagella and fimbriae protein and displayed on the surface of the bacteria. A typical library can combine up to 10^11^ different peptides (Table 3). Hecht and colleagues developed a method for co-expression of combined random peptides with the Aβ42-GFP fusion protein in *E. coli* (Wurth et al., 2002). When the construct is expressed recombinantly in *E. coli*, the Aβ42 sequence and the N-terminal fusion of GFP will cause aggregation and prevent proper folding and chromophore maturation, while the fused GFP can fold and emit bright fluorescence when Aβ42 is prevented from accumulation. Baine et al. (2009) used this method to screen short peptides that can inhibit the aggregation of Aβ42, and proved that the Peptide 2 they screened is one of the few peptides known to decompose preformed Aβ42 fibers. Arslan et al. (2010) predicted that the amphipathic helix could convert Aβ into a natural-like protein and inhibit the initiation of oligomerization and aggregation. They screened a semi-random amphipathic helix sequence library and replaced GFP with YFP to optimize the signal-to-noise ratio further. They also proved that the selected SV111 peptide could induce the natural-like structure in Aβ42 and inhibit the formation of amyloid fibrils. Since bacteria can be modified to incorporate fluorescent labels, another major advantage of bacterial libraries is the ability to use fluorescence activated cell sorting (FACS) library screening, allowing quantification of clone binding. Skretas and colleagues synthesized large combinatorial libraries of macrocyclic molecules in *E. coli* cells, and used flow cytometry to screen cyclic peptides with the ability to rescue pathogenic protein misfolding and aggregation in an ultra-high-throughput manner (Figure 3B; Matis et al., 2017; Delivoria et al., 2019).

**TABLE 3:** Aβ-targeting peptides identified from bacterial-display peptide libraries.

Receptor	Sequence	Library design	References
Aβ fibrils	NGRHVLRPKVQA VRHVLPKVQAPV	FliTrx random peptide library	Schwarzman et al., 2005
Aβ42	GDKAGAEVLAAVKAIKEK (Sv111)	Semi-random Library of Amphipathic Helices	Arslan et al., 2010
Aβ42	Peptide 1A (MSNKGASIGLMAGDVDIADSHS) Peptide 1B (MSNKGASNALMAGDGDIADSHS) Peptide 2 (MQKLDVVAEDAGSNK)	Designed to match the aggregation-prone regions of Aβ42	Baine et al., 2009
Aβ42	Cyclo-TPVWFD; cyclo-TAFDR, cyclo-TAWCR, cyclo-TTWCR, cyclo-TTVDR, cyclo-TTYAR, cyclo-TTTAR, and cyclo-SASPT	Combinatorial libraries of random cyclic tetra-, penta-, and hexapeptides	Matis et al., 2017
Aβ42	Cyclo-CKVWQLL (AβC7-1) cyclo-CRIVPSL (AβC7-14)	Random head-to-tail cyclic heptapeptide library	Delivoria et al., 2019

**FIGURE 4:**
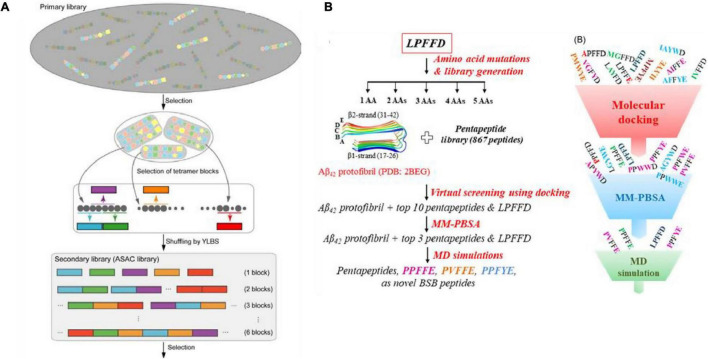
**(A)** Illustration of the designated progressive library method (PLM) (Tsuji-Ueno et al., 2011; Ghimire Gautam et al., 2015); **(B)** schematic illustration of the *in silico* screening workflow for designing novel β-sheet breaker peptides (Shuaib et al., 2019). Reproduced with permission.

The cDNA display library has also been used for the screening of high-affinity Aβ binding peptides. Koichi and colleagues developed a systematic evolution *in vitro* and designated progressive library method (PLM) to obtain high-affinity peptide aptamers, which is to screen a diversified secondary peptide library constructed based on the information obtained from the primary library selection (Figure 4A; Tsuji-Ueno et al., 2011; Ghimire Gautam et al., 2015). In addition, computer screening has gradually begun to be applied in recent years. Based on the known β-sheet disruptor peptide LPFFD, Shuaib et al. (2019) used virtual screening based on molecular docking to identify pentapeptides with stronger binding affinity than LPFFD and utilized the MM-PBSA method to evaluate the binding free energy of the first 10 pentapeptides. Subsequently, molecular dynamics simulations were used to determine that the pentapeptides PPFFE, PVFFE, and PPFYE are potential BSB peptides for destroying the stability of the Aβ42 fibril structure (Figure 4B). Sievers et al. (2011) developed a non-natural D-peptide tau inhibitor (D)-TLKIVW (TLK) through a computational design based on the atomic structure of the tau aggregation motif (VQIVYK), and proved that it could be combined with tau aggregates instead of tau monomers through hydrogen bonding and hydrophobic interaction. Zhu et al. (2021) modified the multivalent TLK peptide to the surface of a multifunctional nanoinhibitor with core hydrophobic PCL and shell hydrophilic PEG. By capturing tau aggregates multivalently, the inhibitor not only effectively inhibits the growth of tau protein aggregates and prevents their spread across cells, but also promotes the proteolytic degradation of tau aggregates, and offers a potent and straightforward way to realize the tau-targeted treatment of AD.

### Peptoid

**FIGURE 5:**
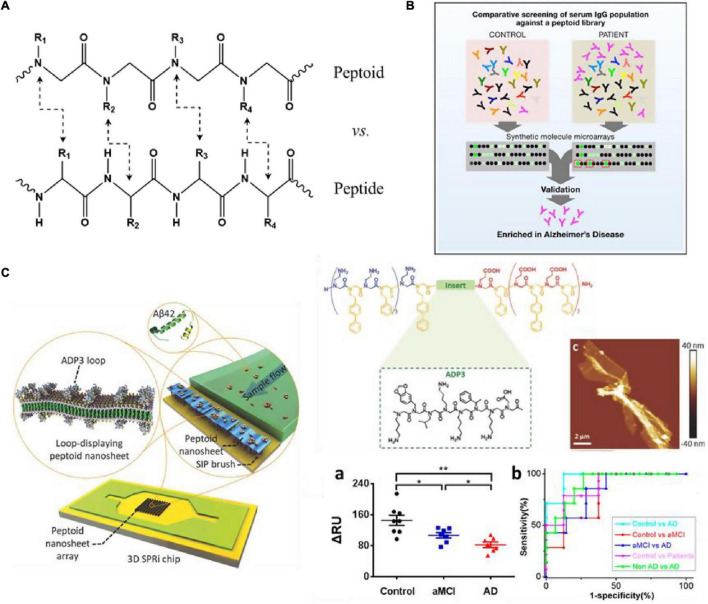
**(A)** Chemical structure comparison of peptide and peptoid; **(B)** schematic overview of the process employed to screen peptoids that bind to antibodies present at higher levels in AD patients (Reddy et al., 2011); **(C)** structure of ADP3 microarray on 3D SPRi chip to develop a rapid, label-free sensor (Zhu et al., 2017; Gao et al., 2020). Reproduced with permission. Significance comparisons between groups were calculated by one-tailed unpaired *t*-test with Welch’s correction. *P < 0.05, **P < 0.01.

In general, most peptides cannot be administered orally, have a short half-life, potential immunogenicity, and poor *in vivo* metabolic stability. Therefore, they are challenging to utilize directly in the biomedical field, often requiring delivery, modification or development of peptide mimics (Patch and Barron, 2002). As a synthetic, convenient, modular peptide mimic, peptoid developed in the late 1980s (Simon et al., 1992), has the same molecular skeleton as the peptide, but its side chain is attached to the amide nitrogen instead of the α-carbon, accompanied by loss of main chain chirality and amide hydrogens, as shown in Figure 5A (Ganesh et al., 2017). The peptide backbone is entirely composed of triamides, and the presentation of peptoid side chains is roughly equidistant, which may allow for an appropriate simulation of the spacing of critical groups of bioactive peptides. Besides, this modification confers protease resistance because natural proteases do not recognize N-substituted amide bonds (Saini and Verma, 2017). The lack of backbone chirality and amide hydrogens avoids the challenges of limiting production and interferes with the secondary structure, conferring much conformational flexibility in the main chain (Sun and Zuckermann, 2013). In the past few decades, the introduction of submonomer method has made the design and synthesis of peptoids more convenient and efficient, making the structure of peptoids flexible and rich in types (Zuckermann et al., 1992). In this strategy, peptoid monomers are synthesized in two steps: acylation reactions with bromoacetic acid followed by nucleophilic displacement of bromine with a primary amine. Coupled to better protease resistance and stability and enhanced pharmacological properties and activity, peptoids for biomedical applications can be an excellent platform as probes and sensors, also has broad application prospects in the field of AD detection and treatment (Vanderstichele and Kodadek, 2014).

2D nanomaterials have attracted widespread attention and research in recent years. The characteristics of engineered ability at the molecular level, chemical diversity, and synthesis flexibility compared to natural proteins and peptides make peptoids seem more suitable to fabricate highly tunable two-dimensional nanostructures through self-assembly (Lau, 2014). Zuckermann et al. (1992) used a water-soluble nanosheet with two molecular layers as an antibody simulation platform. At the gas-liquid interface, the amphiphilic peptoid molecules are tightly arranged to form a monomolecular layer under extrusion conditions (Nam et al., 2010). Then the monomolecular layer collapses toward the liquid surface, so that the hydrophilic part is exposed to the outside and the hydrophobic component is wrapped inside to form a stable Nanosheet structure. Then insert oligopeptide fragments with known recognition characteristics between the peptidomimetic sequences with periodic self-assembly motifs to create a nanosheet with a cyclic structure on the surface (Knight et al., 2015). This structure has better stability and lower cost than antibodies, thus having a good application prospect.

Reddy et al. (2011) used peptoid arrays to screen AD biomarkers, aiming to screen out a large number of synthetic molecules that can target antigen-binding sites without prior knowledge of natural antigens. Through the screening serum samples of six AD patients, six Parkinson’s disease (PD) patients and 6 normal controls, they successfully selected 3 peptoids (ADP1-3) that specifically bind to IgG in AD serum from 4608 peptoids molecules (Figure 5B). And in the screening of 50 patients’ serum samples, these three peptides showed good sensitivity and specificity for the diagnosis of AD (all over 90%). Zhao et al. (2015) combined ADP3 microarray with surface plasmon resonance imaging (SPRi) to develop a rapid and label-free diagnostic method for AD, and demonstrated that high concentrations of ADP3 can recognize AD by binding to Aβ42 in serum. As shown in Figure 5C, they further inserted ADP3 into the amphiphilic peptoid to construct antibody mimics self-assembled peptoid nanosheets, including the surface-exposed Aβ42 recognition loop, as a label-free sensor used to detect AD serum (Zhu et al., 2017). The dense distribution of molecular recognition loops on the sturdy peptoid nanosheet scaffold mimics the structure of antibodies and reduces non-specific binding when detecting multi-component samples, further improving the sensitivity and accuracy of AD serum detection. Gao et al. (2020) used the peptoid nanosheet-SPRi sensor to measure the serum and plasma of patients with aMCI and AD and healthy controls. They proved that each group could be distinguished significantly with high sensitivity and specificity (AUC: 0.8–0.96) (Gao et al., 2020, 2021). This system is exquisitely sensitive, highly specific for AβOs and facilitates detection of biologically-relevant species in a complex matrix. Yazdani et al. (2016) identified a peptoid PD2 that can be useful for the early-stage identification of PD and serve as an indicator of disease severity. Gao et al. (2019) identified a peptoid ASBP-7 that recognizes α-synuclein in serum and provides a method for blood-based PD detection. Author further confirmed that ASBP-7 through specifically binding to α-synuclein in the serum can be effectively distinguished Parkinson’s disease from serum of normal persons.

In addition, Luo et al. (2013) constructed a combinatorial peptide library with a library capacity of 38416 and obtained Aβ42 specific ligand IAM1 and its dimer (IAM1)2. Through dimerization, (IAM1)2 has a higher affinity with Aβ42 than (IAM)1. Both are promising to be used in the development of Aβ42 detection reagents or as lead therapeutic compounds, as they can effectively inhibit the aggregation of Aβ42 and reduce the neurotoxicity caused by Aβ42 *in vivo*.

### Enzyme-Responsive Peptides

In recent years, enzyme-responsive peptides have been served as the “switch” of fluorescent probes as a simple and sensitive fluorescent biosensing platform for enzyme detection. BACE1 catalyzes the hydrolysis of the amyloid precursor protein, which is the rate-determining step of Aβ production; thus, it plays an essential role in the occurrence of AD and is considered a vital AD detection and therapeutic target (Esler and Wolfe, 2001; Luo et al., 2001; Evin et al., 2010). Using peptide substrates to develop a simple and rapid method to detect BACE1 and screen its inhibitors is conducive to AD’s clinical diagnosis and treatment.

The APP Swedish mutation sequence (EVNLDAEF, representing residues 668-675) is a better BACE substrate than natural APP, and the enzyme cleavage between leucine and aspartic acid residues (Folk and Franz, 2010). Zuo et al. (2018) constructed a sensor platform based on WS2 nanosheets and the FAM-labeled peptide substrate, which can be adsorbed on the surface of the WS2 nanosheet to quench its fluorescence. In the presence of BACE1, BACE1 hydrolyzes the peptide substrate to release short FAM-linked peptide fragments, thereby restoring the fluorescent signal. The fluorescence sensing platform can be used to monitor BACE1 with a detection limit of 66 pM and has been proven to be suitable for screening BACE1 inhibitors. Kim S. et al. (2020) constructed a magnetic graphite oxide (MGO) alkenyl FRET biosensor, on which they synthesized an N-terminal FITC-labeled peptide specific to BACE1 (EVNLDA). The resonance energy transfer from FITC to MGO results in the quenching of the fluorescence generated from FITC. The fluorescence is restored after the BACE1 cleavage releases the FITC-peptide fragment. This method could successfully measure BACE1 in the range of 0.125 ng/mL to 1.2 μg/mL and construct imaging probes. Ge et al. (2020) developed a two-photon ratio fluorescent probe for the imaging and sensing of BACE1 in living cells and deep tissues, in which the peptide substrate connects the two-photon donor cyanine derivative (mCyd) with the acceptor Alexa Fluor 633 (AF633). The fluorescence emission ratio of AF633mCyd shows good linearity in the range of 0.1–40.0 nM, and LOD is reduced to 65.3 ± 0.1 pM. They successfully applied the probe to the imaging and sensing of BACE1 in different areas of the brain tissue of AD mice with a depth greater than 300 μm.

## Conclusion and Perspective

In AD, although cerebrospinal fluid biomarkers are the most well-researched and widely accepted, blood biomarkers show greater prospects in the diagnosis of AD. The widespread application of blood biomarkers relies on the development and application of ultra-sensitive detection methods (Karki et al., 2021), so their accurate detection and quantification will greatly facilitate current and future diagnostic and therapeutic efforts. This review introduces the application of peptides or peptide mimics of different sources and functions as identification elements or carriers of biosensors or molecular probes in the highly sensitive detection and diagnosis of AD. The peptide is used as a non-antibody replacement recognition molecule with low immunogenicity, lower manufacturing cost, and easier access to chemical diversity. Also, because of its small size, it has a better ability to pass through the blood-brain barrier. Self-assembled peptides provide enhanced surface area and binding sites to increase the accumulation of signal probes and bind to special peptide domains to display specific functions, serving as a signal amplification strategy in biosensors. A variety of peptide-based biosensors have been shown potential for detecting blood biomarkers due to their pg-level detection sensitivity.

Nevertheless, there has been no success in the clinical translation of these peptide-based approaches, which require further development to meet the needs of early detection and clinical diagnosis. Future studies should consider the following significant items. First, screening for higher-affinity peptides targeting AD biomarkers remains a considerable challenge, with currently the most reliable peptides for biosensors still derived from native ligands. And most of the peptides or peptoids now used in AD detection are for Aβ or Tau-related biomarkers, but studies have shown that new biomarkers are needed to track non-Aβ and non-tau pathology (Park et al., 2020). Second, future detection methods should be established closer to the clinical standards, rigorous control of assay performance is essential (Andreasson et al., 2015). The standard calibration curve and LOD measurements of some approaches are based on a simple system with pure proteins, which does not effectively indicate whether it is still effective on complex fluids. In contrast, others use the standard addition method in which the use of labeled proteins may prevent these studies from comparing to each other. Alternatively, differences between the calibrator and the endogenous analyte can make the specificity of the assay difficult to determine, so it’s usually necessary to investigating the parallelism. The repeatability and reproducibility of the test method should also be checked. Recently, the ultrasensitive immunoassay technique (single-molecule array, Simoa) has achieved great progress in the sensitive detection of plasma A β and p-tau (Chatterjee et al., 2021). However, early and effective diagnosis of AD remains a challenge due to the complexity of AD pathogenesis, which leads to production of several related biomarkers and lack of reproducibility. In addition, the combination of multiple biomarkers can greatly improve diagnostic sensitivity and specificity (Kim K. et al., 2020; Cullen et al., 2021). The challenge is to use peptide ligand with different specificities to develop multiple devices and establish multiple detection of several AD biomarkers in the same sample solution.

Due to the differences in sensitivity and detection thresholds reported in different studies, it is difficult to use the same standard to evaluate. To confirm the validity of AD candidate blood biomarkers, the sampling, processing and analysis methods should be standardized to ensure reproducibility of results while minimizing variability between laboratories. In addition, large samples should be evaluated to assess the concentration range of biomarkers in the clinical routine. With the establishment of more and more AD biomarkers and the development of detection methods, peptide nanomaterials are currently under-utilized in the early diagnosis of AD and its potential warrants further exploration (Scarano et al., 2016). In the future, blood-based biomarkers could potentially be applied to clinical routine practice and point-of-care testing to monitor the AD progress and efficacy of disease therapies in individual patients.

## Author Contributions

YG and ZW wrote the manuscript. ZH revised the manuscript and supervision. All the authors approved the manuscript.

## Conflict of Interest

The authors declare that the research was conducted in the absence of any commercial or financial relationships that could be construed as a potential conflict of interest.

## Publisher’s Note

All claims expressed in this article are solely those of the authors and do not necessarily represent those of their affiliated organizations, or those of the publisher, the editors and the reviewers. Any product that may be evaluated in this article, or claim that may be made by its manufacturer, is not guaranteed or endorsed by the publisher.
