# Corrigendum: Recent Advances in the Application Peptide and Peptoid in Diagnosis Biomarkers of Alzheimer's Disease in Blood

**DOI:** 10.3389/fnmol.2022.865110

**Published:** 2022-02-21

**Authors:** Yuxin Guo, Zhiyuan Hu, Zihua Wang

**Affiliations:** ^1^CAS Key Laboratory of Standardization and Measurement for Nanotechnology, CAS Key Laboratory for Biomedical Effects of Nanomaterials and Nanosafety, CAS Center for Excellence in Nanoscience, National Center for Nanoscience and Technology, Beijing, China; ^2^University of Chinese Academy of Sciences, Beijing, China; ^3^Fujian Provincial Key Laboratory of Brain Aging and Neurodegenerative Diseases, School of Basic Medical Sciences, Fujian Medical University, Fuzhou, China; ^4^School of Nanoscience and Technology, Sino-Danish College, University of Chinese Academy of Sciences, Beijing, China; ^5^School of Chemical Engineering and Pharmacy, Wuhan Institute of Technology, Wuhan, China

**Keywords:** Alzheimer's disease, blood biomarkers, diagnosis, peptide, peptoid

In the published article, there was an error regarding the affiliations for Yuxin Guo. As well as having affiliation 1, they should also have “University of Chinese Academy of Sciences, Beijing, China”.

The authors apologize for this error and state that this does not change the scientific conclusions of the article in any way. The original article has been updated.

## Publisher's Note

All claims expressed in this article are solely those of the authors and do not necessarily represent those of their affiliated organizations, or those of the publisher, the editors and the reviewers. Any product that may be evaluated in this article, or claim that may be made by its manufacturer, is not guaranteed or endorsed by the publisher.

